# The Islamic principle of *ḥifẓ al-nafs* (protection of life) and COVID-19 in Indonesia: A case study of nurul iman mosque of Bengkulu city

**DOI:** 10.1016/j.heliyon.2021.e07541

**Published:** 2021-07-15

**Authors:** Moh Dahlan, Mohammad Reevany Bustami, Makmur, Siti Mas'ulah

**Affiliations:** a IAIN Bengkulu, Indonesia; b CenPRIS Universiti Sains Malaysia, Malaysia; c IAIN Bengkulu/ STIESNU Bengkulu, Indonesia

**Keywords:** Protection of life, Covid-19, Worship, Mosque imams, Mosque congregations

## Abstract

**Background:**

The act of worshipping in mosque is often not only considered to be one of the factors causing the transmission of coronavirus disease 2019 (covid-19), but also a contributing factor to health protocol violations. Against this backdrop, the purpose of this paper is to examine the application of the Islamic principle of *ḥifẓ al-nafs* (protection of life) in the practice of worshipping at the Nurul Iman mosque as it relates to the prevention of transmission of covid-19.

**Methods:**

This research uses a sociology of law perspective to the connection and the dynamics between the adoption of the principle of *ḥifẓ al-nafs* and the implementation of worship at the Nurul Iman mosque. As a case study, the data collection technique used includes interviews, observation, documentation reviews, and triangulation techniques, while the analysis technique used content analysis techniques.

**Results:**

The application of the principle of *ḥifẓ al-nafs* (protection of life) in the practices of worship at the Nurul Iman mosque have proven to be in accordance with health protocols and at the same time all members of the mosque congregation have been free from the transmission of covid-19. From an Islamic needs framework perspective, in the context of Covid-19 pandemic, the application of the *ḥifẓ al-nafs* principle at this mosque is a considered primary necessity (*ḍarūriyyāt*) in that it protects life while providing spiritual continuity of collective religious worshipping, hence integrating the community together within a health and sanctity of life paradigm during this challenging time. Equally important, this research also challenges the thesis of the direct linkage between worshipping in mosque and Covid-19 transmission.

**Conclusion:**

the application of the principle of *ḥifẓ al-nafs* in the practices of worship in mosque has demonstrably shown that the congragants levels of health consciousness has increased and that they have also been kept safe. In essence, the effective contextualization of Islamic principle is able to provide the twin benefits of health and spirituality.

## 1 Introduction

The role of every religion cannot be denied in maintaining the safety of human life (Bello, 2014; Hermawan, 2014; Layish, 2014; Seligman and Casanova, 1994). Likewise, this is true for Islam. The sanctity of human life is held at the utmost position in Islamic religious norms as prescribed by *maqāṣid al-syarī’ah* (objectives or contextual-transcendental intent of sharia) which teaches the principle of protection of life (*ḥifẓ al-nafs*) (Fikri Zuhriyah, 2012; Mudzhar, 2013, 2014; Rambe, 2016; Wahid, 2011).

As human life continues to develop and evolve, *ijtihād* (religious judgement or ruling especially responding to social context or phenomena) has an important role in maintaining the safety of human life (Mudzhar, 2013). In upholding the contextual importance of *ijtihād*, a hadith (saying of the Holy Prophet) conveys that “With regards to the affairs of your world, it is you who have a better understanding.” (*Ḥadīs Riwāyat Muslim*)”. It is within this contextual guidance, in responding to the dynamics of human social life, Islamic religious norms provide space for the freedom to carry out *ijtihād*, but in matters of worship, Islamic religious norms also provide parameters to it. However, when the dimension of worship as a divine vertical relationship with God (*ḥablum min Allāh*) pertains to human dimension, which a horizontal social relationship, it is also influenced by the human contexts. In other words, the human dynamics (as a horizontal relationship) and the worshipping of Allah (as a vertical relationship) converge into two inseparable sides of the coin. As an example, sharia (the rule) of *wudhu'* (taking ablution), is not only to fulfill the purity requirements for praying, but also to maintain the cleanliness of the human body in order to ensure the safety of human life (*ḥifẓ al-nafs*) (Ahmad, 2014; Maawiyah, 2016).

Regarding the application of the principle of *ḥifz al-nafs* during the Covid-19 epidemic, Muslims in various parts of the world carried out various efforts, including one by closing religious activities in mosque, namely not holding Friday prayers and congregational prayers and other worship in mosque to prevent a meeting of many people that can cause the transmission of covid-19, even some Islamic scholars argue that when an area is in a red zone (i.e. high potential for covid-19 transmission), religious activities in mosque must be closed or suspended until normal conditions (Fajriah, 2020; Fauziah, 2020; Qotadah, 2020), so that a religious group or mosque that is still holding worship at the mosque is considered to be able to cause the spread of covid-19 and violate health protocols. With regards to the policy of determining the red zone, the Nurul Iman mosque of Pagar Dewa in Bengkulu City is a mosque that is different from other mosque policies. Nurul Iman mosque continues to hold worship at the mosque by upholding the principle of *ḥifẓ al-nafs* in accordance with the health protocol policy.

At present, studies on the practices of worship in mosque (or house of worship) tend to examine either from the perspective of suspension of worship or from the perspective of the causes of covid-19 transmission. This can be illustrated in the following studies and scientific reports: First, a research by Megatsari et al. (2020) which stated that some religious groups still gather to perform religious rituals in which some new cases can potentially occur. *Second*, Carminanda (2020a) who explained that the implementation of congregational worship had led to the spread of covid-19 for congregants at the At-Taqwa Grand Mosque in Bengkulu city (Carminanda, 2020b). *Third*, Tsani (2020) who explained that holding the general recitation (*tabligh akbar*) at the Mosque Jamek Sri Petaling, Kuala Lumpur, Malaysia had caused the spread of Covid-19. Fourth, research Sadiah et al. (2020) who explained that the implementation of congregational worship at the Al-Muhajirin Mosque in Bandung Regency was stopped in order to prevent the spread of covid-19. Fifth, the application of *maqāṣid al-syarī’ah* is used to suspend the implementation of worship in mosque (Qotadah, 2020). Sixth, the limitation of social gatherings has changed the paradigm of conducting worship in churches, so that the implementation of worship is also carried out online in each person's home (Widjaja et al., 2020).

The purpose of this paper is to complement the shortcomings of previous studies by analyzing how the application of the *ḥifẓ al-nafs* principle in the implementation of worship at the Nurul Iman mosque not only preventing the transmission of covid-19 but also complying with health protocols. The evidence of the application of the *ḥifẓ al-nafs* principle in the implementation of worship at the Nurul Iman mosque which is oriented towards preventing the transmission of covid-19 can be an important lesson for the implementation of worship in other mosque in particular and also for the implementation of worship in places of worship in general. This paper is based on the argument that the implementation of worship in mosque which has promised the safety of human life (*ḥifẓ al-nafs*) is not only considered a factor causing the transmission of covid-19, but also a factor that causes violations of health protocols, so its implementation must be postponed, even stopped. Based on this description, the following research questions were formulated: how is the implementation of worship at the Nurul Iman mosque of Pagar Dewa in Bengkulu City? how is the way to apply the principle of *ḥifẓ al-nafs* in preventing the spread of covid-19 at the Nurul Iman mosque of Pagar Dewa in Bengkulu City?

## 2 Theory

### 2.1 Coronavirus disease 2019

Coronavirus disease 2019 (covid-19) has a faster spread rate, in fact more than one hundred countries have implemented lockdowns on a national scale (national quarantine) and also partially on a local scale (local quarantine). The Indonesian government has also implemented local scale quarantine. As of April 25, 2020, Covid-19 cases have reached 5,923 people in Indonesia. Meanwhile, on a global scale, Covid-19 cases have reached 2,167,398 people (BBC, 2020). On April 16, 2020, the status of People Under Monitoring (*Orang Dalam Pengawasan*/ODP) totaled 554 people and four confirmed cases of Covid-19 were in Bengkulu Province. On May 29, 2020, confirmed cases of covid-19 increased to 71 people in Bengkulu Province (Carminanda, 2020a; Dong et al. 2020; Gabrillin 2020; Riou and Althaus 2020; Singhal 2020).

### 2.2 The concept of *maqāṣid al-syarī’ah*

The concept of *maqāṣid al-syarī’ah* is a concept that determines the objectives of the development of Islamic religious norms (laws) in maintaining the safety of human life by bringing benefits to life and rejecting the death of life. In Islamic religious norms, the concept of *maqāṣid al-syarī’ah* is a parameter of *ijtihād* in formulating and determining legal provisions that will be applied to humans. In this case, *maqāṣid al-syarī’ah* has four elements, namely the purpose or intended rationale of God in establishing Islamic religious norms (Islamic law) for particular contexts, God's aim in establishing understandable Islamic religious norms, God's purpose in mandating *mukallaf* (mature and mentally competent human beings) who understand the demands and the will of Islamic religious norms, and the purpose of God's mandate to *mukallaf* to submit to Islamic religious norms.

These elements serve as guiding principles lines for *ijtihād* in formulating Islamic religious norms vis-à-vis their needs. These guidelines are differentiated based on the types of human needs. The first is primary needs (*ḍarūriyyāt*). These are the necessities of human life that must be met. If men do not fulfill these needs, they will suffer to a point possible self-destruction. The next element is secondary needs (*hājiyyāt*). The guiding principle is that secondary needs are necessities of human life that must be fulfilled. However, unlike the primary needs (*ḍarūriyyāt*), if humans do not fulfill them, they will have difficulties in life but not necessarily leading to destruction. The third element, complementary needs (*taḥsīniyyāt*), is a type of necessity of human life that needs to be met, but if humans do not fulfill them, then he will have little or no trouble or difficulties, relative to the second element.

With reference to primary needs, *maqāṣid al-syarī’ah* stems from five main elements, namely *ḥifẓ al-dīn* (protection of religion), *ḥifẓ al-nafs* (protection of life), *ḥifẓ al- 'aql* (protection of mind), *ḥifẓ al-nasl* (protection of the offspring), and *hifẓ al-māl* (protection of property). In essence, *maqāṣid al-syarī’ah* has a dimension of worship (divinity) which is to be attained through supra-rational (*ta'abbudi*) dimension as well as a human dimension reached through rationally (*ta'aqquli*). In the context of the human dimension, the implementation of the principles of *ḥifẓ al-nafs* becomes guiding framework for worshipping in that the acts of worship which is a practice of the *ḥifẓ al-dīn* (protection of religion) principle is to a large extent determined by the *ḥifẓ al-nafs* (protection of life) principle. Extrapolating form this, the logic of Islamic religious norms places a high priority on protection of life which in turn may supercede and shape the nature of worship (AUDA, 2019; Duderija, 2014; Jamaa, 2011a; Kamali, 1998; Nur Wahyudi, 2015; Opwis, 2005; Sidiq, 2017; Susanto, 2017).

In ensuring the safety of human life during the Covid-19 pandemic, the application of the *ḥifẓ al-nafs* principle which represents the government's health protocol policy can be described as follows: First, shortening the period of encounter between humans. Second, keeping a safe distance between individuals and not making direct physical contact. Third, wearing of mask. Fourth, maintaining hygene for the individuals as well as the surrounding environment. Fifth, sustaining and strengthening of mental and physical health. This is also in accordance with the decree of the fatwa of the Indonesian Ulema Council (*Majlis Ulama Indonesia*/MUI) Number 31 of 2020 (Athena et al., 2020; Hafil, 2020; Jamaa, 2011b; Kedunggupit, 2020; Syandri and Akbar, 2020; World Health Organization, 2020; Zahrah, 1997).

### 2.3 Implementation of worship at the Mosque

In carrying out the health protocol policy, the fatwa of the Indonesian Ulema Council Number 14 of 2020 provided practical guidelines for the implementation of worship in mosque with the following provisions: First, people exposed to Covid-19 are required to self-isolate. Second, a healthy person must pay attention to the following matters: (a) if a person is in an area with a high potential for infection, he may leave Friday prayers and replace them with Zuhur prayers; and (b) if a person is in an area with low transmission potential, then he is still obliged to perform the obligation of praying by implementing health protocols. Third, if the spread of covid-19 is not controlled in an area, then Muslims are not allowed to hold Friday prayers in that area and are obliged to replace them with Zuhur prayer. Thus, the decision of the Indonesian Ulema Council has provided an alternative option in carrying out legal *ijtihād* in accordance with the dynamics of Muslim social life (Hafil, 2020; Jamaa, 2011b; Zahrah, 1997).

As for the sholat prayer services, a balanced or an integrated solution is required, while upholding Islamic religious norms. First, a place of prayer (such as a mosque) must be clean and free from *najis* (impurities), as anything that is considered impure will nullify acts certain types of worship, which includes the sholat prayers. If the place of prayer is unclean, it must be cleaned and purified by using uncontaminated water based on religious standards and requirements (that the water must be clean/pure and has properties to cleanse/purify other entities). Second, the clothes worn by *muṣalli* (people who pray or perform sholat prayers) must be clean and pure from all types of impurities. Third, the body of *muṣalli* must be hygenic and cleansed from all forms of impurity and *hadaṡ*. *Ḥadaṡ* is an impure condition for Muslims which causes them not to fulfill their obligations of worship. *Ḥadaṡ* consists of two kinds.

The first is “*minor ḥadaṡ*” which requires Muslims to perform ablution (by using pure and uncontaminated water fulfilling the religious definition pure and able to purify) which is done by fulfilling the main elements of Islamic ablution, namely the *niat* (intention) of ablution, washing the face, washing the hands to the elbows, washing forehead and parts of hair near the forehead, and washing the feet to the ankles. In addition, it is also part of Prophet Muhammad's practice *(sunnah)* to also wash hands until they are clean, inhale water sufficiently deep into the nose and then release it, brush teeth, and wash fingers and parts in between as well as toes. The second is “*major ḥadaṡ*” which requires Muslims to take a bath using uncontaminated water while following the requirements of Islamic purification to cleanse from a major ḥadaṡ (Ahmad, 2014; Al-Haitamī and bin Ḥajar, n.d.).

## 3 Method

### 3.1 Research design and data

This research was conducted in Nurul Iman mosque due to in the implementation of worship, Nurul Iman mosque has regulated and organized the worship activities in accordance with the *ḥifẓ al-nafs* principle in the midst of the Covid-19 pandemic (Ausrianti et al., 2020; Jamaa, 2011a; Mosqueuna.com, 2019; Yani, 2000). This research is field case study which uses sociology of law approach to provide a explanatory analysis of the dialectical relationship between the human dimension (*ḥifẓ al-nafs*) and the dimensions of worship (*ḥifẓ al-dīn*) in the midst of the Covid-19 pandemic (Mudzhar, 2013), So that the primary data sources are the findings from the interviews with mosque administrators, mosque Imams, mosque congregations, and field observations in the dynamics of worship at Nurul Iman mosque, while secondary data are derived from library sources which include literature as well as reports on the principle of *ḥifẓ al-nafs*, the fatwa of the Indonesian Ulema Council, worshipping, covid-19 and other related data.

### 3.2 Source of the data

The sources of this research data included the interviews with mosque administrators, Imams and members of the mosque congregations. The aim is to obtain information useful for exploring and understanding the reasons for the implementation of worship incorporating the principle of *ḥifẓ al-nafs* (protection of life) at Nurul Iman mosque. This is further strengthen by the field observations to corroborate interviews with objective empirical evidence in the field. The library sources are the basis for conducting this research and also instrumental in supporting the ontological validity of this research.

### 3.3 Data collection technique and data analysis

In carrying out and corroborating the data collection, the research has employed four techniques. *First*, the in-depth interview technique was used to conduct face-to-face interviews with informants. *Second*, the research also deployed observation technique to make direct field observations about the implementation of worship in mosque. *Third*, in order to corroborate, the research carried out documentation review on secondary data in the form of written sources, mosque management structure charts or other related information. *Fourth*, triangulation techniques were used to collect the data by cross-checking and validating research data. The content analysis technique was used to construct and understand the meaning of the data collected both from library and field sources to describe and understand the implementation of worship at mosque as an effort to prevent the transmission of Covid-19.

### 3.4 Ethical approval committee

In this article, the authors stated that the ethical approval, documentation the full name of the approving ethical committee, and the informations were obtained for this article from all participants for the interview process.

## 4 Results

### 4.1 Implementation of worship at Nurul Iman mosque, Pagar Dewa village, Bengkulu City

Indonesian society is a pluralistic society (Ardi and Budiarti, 2020). Likewise, the Bengkulu community is a plural society living in an isolated and poorest area on the island of Sumatera. In terms of ethnicity, Bengkulu people consist of the Serawai tribe from South Bengkulu Regency, Seluma Regency, and Kaur Regency, the Lembak tribe from the cities of Bengkulu and Central Bengkulu, and the Rejang tribe from the people of North Bengkulu, Rejang Lebong and Lebong, and immigrant tribes originating from the Javanese, Bugis, Minangkabau, and other tribes. Meanwhile, from a religious perspective, the people of Bengkulu city mostly embraced Islam and a small portion who embraced Christianity, Catholicism, Hinduism, Buddhism, and Confucianism (Shannon et al., 2011). Although the people of Bengkulu city accommodate various ethnic groups and followers of religions, the people are still in harmony with a high religious spirit, so even though the city of Bengkulu has been designated as a red zone (Carminanda, 2020c), Nurul Iman mosque continues to carry out congregational worship by applying the principle of *ḥifẓ al-nafs* (protection of life) (Observation, April 20, 2020) (see Table 1).

**Table 1:** The Organizational Structure of Nurul Iman mosque.

No	Title Name		Name of Mosque Management	
1	Advisors		1. Head of Pagar Dewa Urban Village 2. Head of RW 02 of Pagar Dewa 3. Head of RT 02, 10, 11, and 12 RW 02 of Pagar Dewa 4. H. Yuhanis Akbar 5. H. A. Majid Roantin 6. Kontras Musa, S.Sos, MM	
2	General Chairman		Drs. H Rizkan A Rahman, M.Pd.	
	Chairman I		H. M. Arzum Anwar, SH	
	Chaiman II		Drs M Djufri Ismail	
3	Secretary		Jayan Asmudi, S.Ag, M.H.I.	
	Vice Secretary		Aceng Sirajuddin	
4	Treasurer		H. M. Imron	
5	Imam Mosque I		H. Yuhanis Akbar	
	Imam Mosque II		Su'ah Azhari	
6	Preacher		H. Mahmudah, M.H.I.	
7	Gurim		Managers of Nurul Iman mosque	
8	Sections			
	a	Worship and Spirituality Section	Coordinator: Arifai Anwar	
			Member:	1). Musmulyadi, M.Pd
				2). Drs. Sazili, M.Pd.
	b	Islamic Holiday Commemoration Section	Coordinator: Komaruddin, SE	
			Member:	1). H. Marzuki, S.Pd.
				2). Ali Sumarna
	c	Education and Arts Section	Coordinator: Drs. M Yapin Bakar	
			Member:	1). Juharmadi, S.Pd.
				2). Drs. H.M. Dahlan
	d	Public Relations and Publications Section	Coordinator: IB Sopyan	
			Member:	1) Rahmat Saputra, S.Pd.I.
				2) Seluhin
	e	Fund Raising Section	Coordinator: H. M. Tobil Ahmad, S.Pd.	
			Member:	1) H. M. Selamat Solah, BE
				2) Dahirman, S.Pd.
				3) Samsul Hadi, S.T.

**Figure 1:**
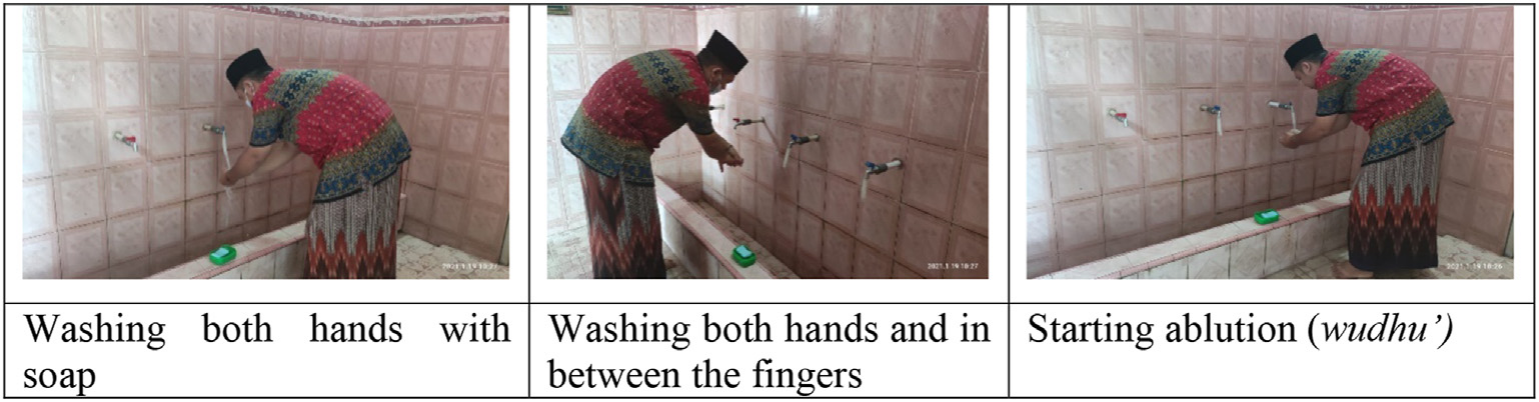
The process of washing hand and starting the ablution.

In terms of congregation, Nurul Iman mosque is not homogeneous because the congregation is not only from the environment around the mosque but also from employees, businessmen and company employees in the Pagar Dewa village, Selebar district of Bengkulu city. Regarding the requirements for the implementation of prayer, Nurul Iman mosque always explained the importance of working on the main elements of *wudhu’ (taking ablution)* until its recommendations were perfect. Therefore, the residents of Nurul Iman mosque performed the basic elements of *wudhu’* using holy water which is done by washing the face, washing the hands to the elbows, washing some of the hair, and washing the feet to the ankles. In addition to working on these basic elements, the mosque's residents also do the recommendation in ablution ', namely brushing teeth, washing both hands to the wrists before *wudhu’*, washing both ears, washing the limbs three times except the head, washing the toes and fingers by interrupting, rinsing their mouths, and washing the living holes. Besides this, *muṣallis* have also been encouraged and are even obliged to take a bath to get rid of *major hadaṡ* (Putra, 2020) (see Figure 1).

In accordance with Rizkan A Rahman's statement (2020), in the implementation of the five daily prayers at the mosque, after reading surah al-fatihah, the mosque imam reads the letters of the Qur'an, namely surah Luqman starting from verse 12 to verse 19, Surah al- Baqarah from verse 1 to verse 20, Surah Ali Imran from verses 102 to 109, Surah al-A'lā which consists of 19 verses, Surah al-Ghāsyaiyyah which consists of 26 verses, Surah al-Ḍuhā which consists of 11 verses, surah al-'Alaq which consists of 19 verses, surah al-takāsur which consists of 8 verses, and surah al-zilzāl which consists of 8 verses, but when the Covid-19 pandemic hit the people of Bengkulu city, the *Imam* (leader) of the mosque then read the *surah* of the Qur'an, including surah Luqman from verse 12 to 14, surah al-Baqarah starting from verse 1 to verse 5, surah Ali Imran from verse 102 to verse 104, surah al-'aṣr which consists of 3 verses, surah al-Fail which consists of 5 verses, surah Qurasy which consists of 5 verses, surah al-Kauṡar which consists of 3 verses, surah al-Kāfirūn which consists of 6 verses, surah al-Naṣr which consists of 3 verses, surah al-Lahab which consists of 5 verses, surah al-Ikhlāṣ which consists of 4 verses, surah al-Falaq which consists of 5 verses, surah al-Nās which consists of 6 verses, and surah al-Taubah verses 28 to 29 (see Tables 2 and 3).

**Table 2:** The Imams of Mosque read the longer verses before Covid-19 pandemic.

NO	NAME	VERSES
01	Surah Luqman	From the 12^th^ verses to the 19^th^
02	Surah al-Baqarah	From the 1^st^ verses to the 20^th^
03	Surah Ali Imran	From the 102^nd^ verses to the 109^th^
04	Surah al-A'lā	From the 1^st^ verses to the 19^th^
05	Surah al-Ghāsyaiyyah	From the 1^st^ verses to the 26^th^
04	Surah al- Ḍuhā	From the 1^st^ verses to the 11^th^
05	Surah al-‘Alaq	From the 1^st^ verses to the 19^th^
06	surah al-takāsur	From the 1^st^ verses to the 8^th^
07	surah al-zilzāl	From the 1^st^ verses to the 8^th^

**Table 3:** The Imams of Mosque read the shorter verses after Covid-19 pandemic.

NO	NAME	Verses
01	Surah Luqman	From the 1^st^ verses to the 14^th^
02	Surah al-Baqarah	From the 1^st^ verses to the 5^th^
03	Surah Ali Imran	From the 102^nd^ verses to the 104^th^
04	surah al-‘aṣr	From the 1^st^ verses to the 3^rd^
05	surah qurasy	From the 1^st^ verses to the 4^th^
06	surah al-kauṡar	From the 1^st^ verses to the 3^rd^
07	surah al-kāfirūn	From the 1^st^ verses to the 6^th^
08	surah al-naṣr	From the 1^st^ verses to the 3^rd^
09	surah al-lahab	From the 1^st^ verses to the 5^th^
10	surah al-ikhlāṣ	From the 1^st^ verses to the 4^th^
11	surah al-falaq	From the 1^st^ verses to the 5^th^
12	surah al-nās	From the 1^st^ verses to the 6^th^
13	Surah al-taubah	From the 128^th^ verses to the 129^th^

In accordance with Ifansyah Putra's statement (2020/2021), mosque residents have generally performed *sunnah qabliyah* prayers (sunnah prayer before performing the five obligatory prayers) and *sunnah ba'diyah* prayers (sunnah prayer after performing the five obligatory prayers) at the Nurul Iman mosque by reading surah al-Fatihah and thereafter then reading the letters of the Qur'an such as surah al-takāsur, surah al-'ādiyat, surah al-humazah, surah al-mā'ūn and other longer letters, but since the Covid pandemic -19 hit the city of Bengkulu, after reading al-fatihah, they generally read al-lahab, al-ikhlāṣ, al-falaq, and al-nās (see Tables 4 and 5).

**Table 4:** Mosque congregation read the longer verses before Covid-19 pandemic.

NO	NAME	Verses
01	surah al-takāsur	From the 1^st^ verses to the 8^th^
02	surah al-humazah	From the 1^st^ verses to the 9^th^
03	surah al-mā’ūn	From the 1^st^ verses to the 7^th^
04	surah al-‘ādiyat	From the 1^st^ verses to the 11^th^

**Table 5:** Mosque congregations read the shorter verses after Covid-19 pandemic.

NO	NAME	Verses
01	surah al-lahab	From the 1^st^ verses to the 5^th^
02	surah al-ikhlāṣ	From the 1^st^ verses to the 4^th^
03	surah al-falaq	From the 1^st^ verses to the 5^th^
04	surah al-nās	From the 1^st^ verses to the 6^th^

Regarding the spread of the Covid-19 pandemic, Musmulyadi (2020/2021) said that since the Covid-19 pandemic occurred, the imam of mosque has performed *dhikr* after the five daily prayers with a shorter duration than before. If before the Covid-19 pandemic, the Imam recites *dhikr* (spiritual chantings) with a longer duration which ends with a prayer for the safety of the world and the hereafter as in Table 6, but after the Covid-19 pandemic, the Imam reads a shorter *dhikr* with *istigfār* (request for forgiveness to God) three times and after that pray for the safety of the world and the hereafter and a prayer to reject *balā'* (tragedies and misfortunes), namely prayer to ask for God's protection from all kinds of trials or disasters such as the threat of the Covid-19 pandemic as shown in Table 7.

**Table 6:** Prayer of the imams of Mosque before Covid-19.

No	Number of readings	Reading ritual *dhikr*
01	3 x	أَسْتَغْفِرُ اللَّهَ الْعَظِيمَ الَّذِي لَا إِلَهَ إِلَّا هُوَ الْحَيَّ الْقَيُّومَ وَأَتُوبُ إِلَيْه
02	3 x	لَاإِلَهَ إِلَّا اللهُ وَحْدَهُ لَا شَرِيْكَ لَهُ، لَهُ الْمُلْكُ وَلَهُ الْحَمْدُ يُحْيِيْ وَيُمِيْتُ وَهُوَ عَلَى كُلِّ شَيْئٍ قَدِيْرٌ
03	1 x	اللَّهُ لَا إِلَهَ إِلَّا هُوَ الْحَيُّ الْقَيُّومُ لَا تَأْخُذُهُ سِنَةٌ وَلَا نَوْمٌ لَهُ مَا فِي السَّمَوَاتِ وَمَا فِي الْأَرْضِ مَنْ ذَا الَّذِي يَشْفَعُ عِنْدَهُ إِلَّا بِإِذْنِهِ يَعْلَمُ مَا بَيْنَ أَيْدِيهِمْوَمَا خَلْفَهُمْ وَلَا يُحِيطُونَ بِشَيْءٍ مِنْ عِلْمِهِ إِلَّا بِمَا شَاءَ وَسِعَ كُرْسِيُّهُ السَّمَوَاتِ وَالْأَرْضَ وَلَا يَئُودُهُ حِفْظُهُمَا وَهُوَ الْعَلِيُّ الْعَظِيمُ
04	33 x	سُبْحَانَ ٱللَّٰهِ
05	33 x	ٱلْحَمْدُ لِلَّٰهِ
06	33 x	ٱللَّٰهُ أَكْبَرُ
07	1 x	Reciting prayers for the salvation of the world and the hereafter

**Table 7:** Prayer of the Imams of Mosque after Covid-19.

No	Number of readings	Reading ritual *dhikr*
01	3 x	أَسْتَغْفِرُ اللَّهَ الْعَظِيمَ الَّذِي لَا إِلَهَ إِلَّا هُوَ الْحَيَّ الْقَيُّومَ وَأَتُوبُ إِلَيْه
07	1 x	Reciting prayers for the salvation of the world and the hereafter

**Figure 2:**
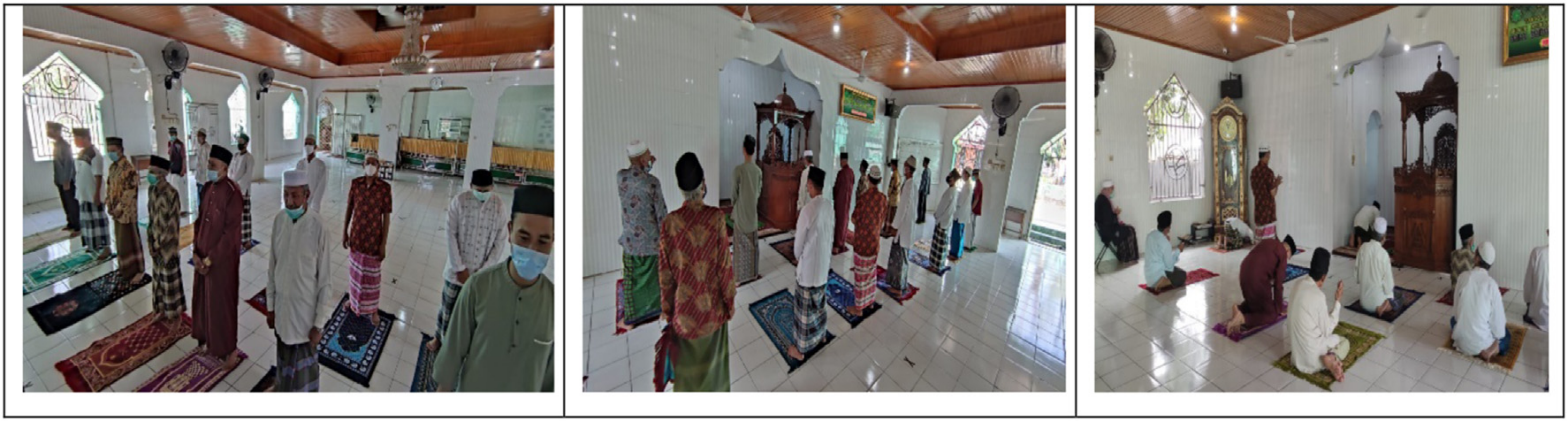
Implementation of Friday prayers and *Dhikr* at the Nurul Iman mosque.

Nurul Iman mosque hold prayer services at a distance among the congregations, either to the side, to the front or to the back. The mosque applies the discipline of performing the prayers in a disciplined manner. Therefore, in performing the *sunnah qabliyah* and *sunnah ba'diyah* prayers, the mosque congregation did both of them with a longer distance among the congregations. This is done because the implementation of the sunnah prayer can be done by moving from the original place when performing the five daily prayers. In addition, the Imams of mosque and congregation of the Nurul Iman mosque also wear masks when they performing the five daily prayers or Friday prayers (Observation 15 April 2020) (see Figure 2).

In accordance with the statement of Akbar (2020), the congregation of the Nurul Iman mosque also performed prayers while practicing physical distance among the members of congregation. The distance among the congregation provided comfort for the worshippers performing their prayers. The practice of maintaining physical distance among congregations has been in effect since the Covid-19 pandemic hit the city of Bengkulu which was marked by the presence of a Covid-19 patient who died in Bengkulu City. The imam of mosque also provided education and Islamic religious guidance to mosque worshipers about the importance of implementing the *ḥifẓ al-nafs* principle as a primary religious obligation in order to increase faith, piety and to overcome anxiety as well as to prevent from the Covid-19 transmission.

### 4.2 Application of the principle of ḥifẓ al-nafs in preventing the spread of the Covid-19 pandemic at Nurul Iman mosque

The difference in the views of the mosque congregation in carrying out their worship at the mosque did occur but did not lead to conflict. They can perform their worship properly and freely based on their beliefs, so that some mosque congregations worshipped in their respective homes and some worshipped in mosque. Before Covid-19 pandemic, there were between 50 and 70 mosque worshipers in the mosque, but when the Covid-19 pandemic hit the city of Bengkulu, the worshipers at the mosque were between 14 and 25 people. For congregation who carry out worship in mosque, praying five times a day was an obligation for every Muslim if it is carried out in congregation at the mosque, then the value of merit and kindness is more (Ahmad, 2020).

Meanwhile, the leadership of the Imam is of the view that obliged to serve congregations who want to carry out congregational worship at the mosque, so that all imams took turns leading the implementation of congregational prayers at the mosque. In addition, the Imam of mosque also guided the mosque congregation to be vigilant and to be careful in carrying out worship in the mosque so that the threat of the Covid-19 pandemic can be avoided (Akbar, 2020).

**Figure 3:**
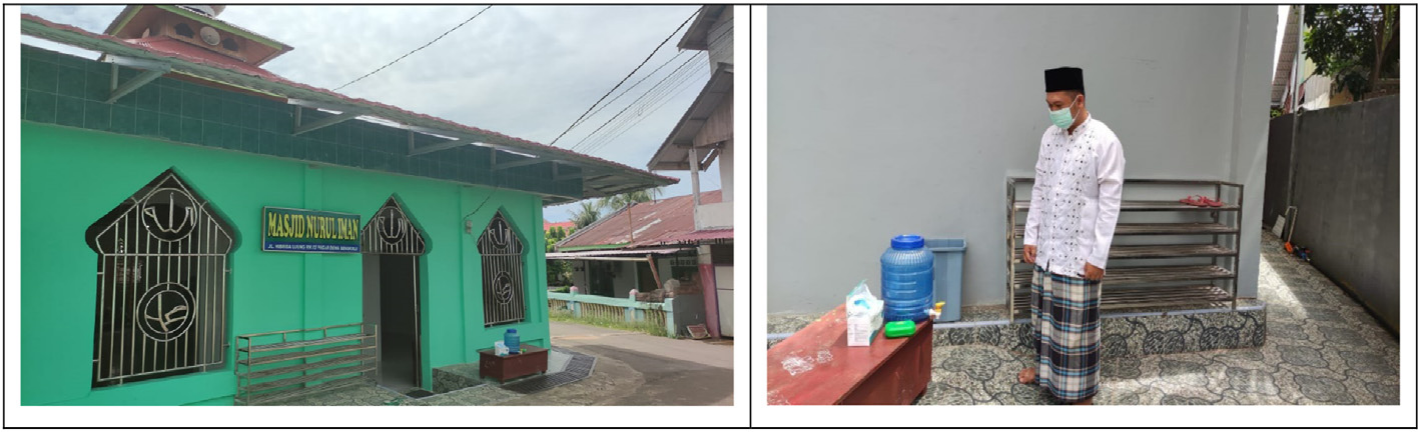
Place for hands washing and masks.

In an effort to avoid the threat of the Covid-19 pandemic, Nurul Iman mosque congregation washed their hands when they are about to enter the prayer hall inside the mosque. They washed their hands in an orderly manner and took turns doing it. They did not have any objection to this practice, in fact they reportedly feel safer from the threat of the Covid-19 pandemic. For mosque congregants, the discipline of washing hands was basically not new in Islamic religious norms. This obligatory tradition is basically a part of Islamic religious practice when someone wants to perform ablution (purify from a small/minor *hadaṡ*). Meanwhile, having *wudhu’* (taking ablution) is one of the religious requirements for performing the sholat prayers (Mahmudah, 2020) (see Figure 3).

**Figure 4:**
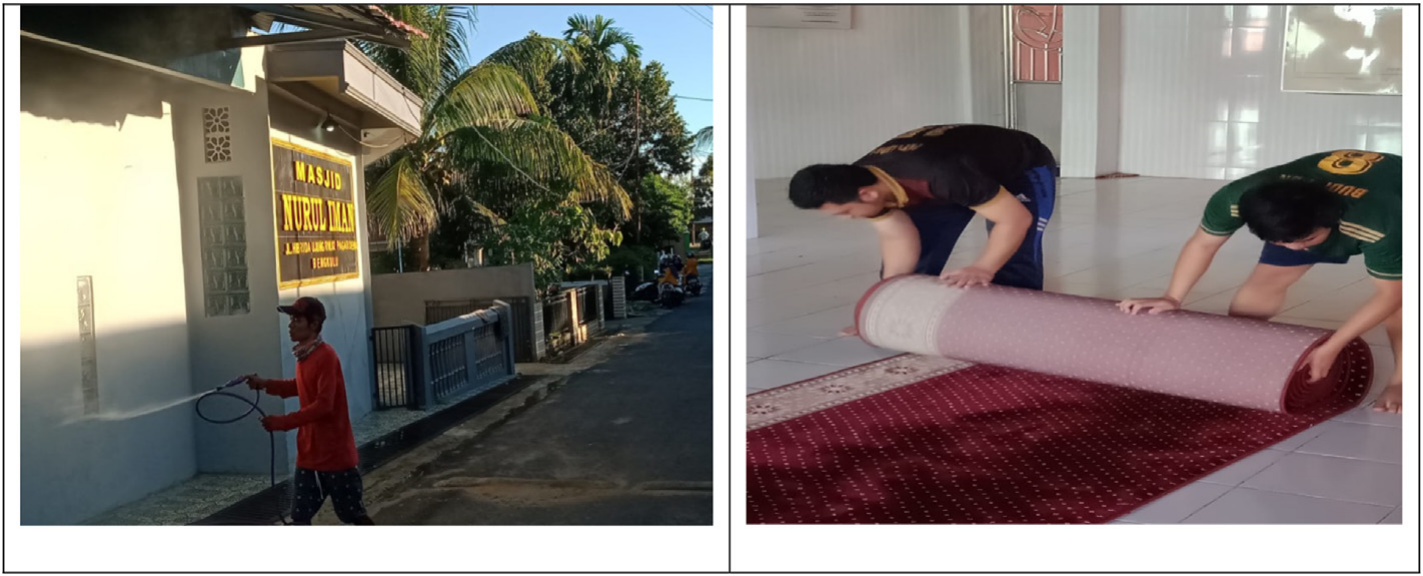
The mosque congregants ensuring cleanliness and spraying disinfectants at Nurul Iman mosque.

In carrying out worship at the mosque, Nurul Iman mosque has prepared a place to wash hands in order to maintain cleanliness, purity and health. The hand washing place is outside of the mosque. Nurul Iman mosque also prepared masks for worshipers who may not have one. In addition, the mosque congregants are also given an understanding of the need to always wear masks, observe a safe distance (physical distancing) among members of the congregation, avoid shaking hands, regularly wash their hands, maintain body condition, wear clean and holy clothes, maintain cleanliness and body health, and maintain the cleanliness and health of the mosque environment (Ahmad, 2020) (see Figure 4).

In accordance with the statement of Azhari (2020), Imam of the mosque also advises the mosque congregation to regularly clean the mosque environment. The cleaning of mosque environment is carried out so that the mosque remains hygenic, spiritually pure, neat and healthy. In maintaining the state of hygiene of the mosque environment, the Imam also regularly briefs the mosque congregations to systematically carry out cleaning activities and spraying of disinfectants. This is an organized effort to prevent and minimize harm by maintaining the safety and hygene of, oneself, one's family and the environment of the mosque, while adhering to government policies and implementing healthy lifestyles.

## 4 Discussions

A pluralistic society, Bengkulu, as a society, generally portrays harmonious characteristics. In line with the research findings of Abdul Hamid (2016), the harmonious nature of the community has become the basic capital in building togetherness vis-à-vis facing various societal problems including the Covid-19 pandemic. Even though the people of Bengkulu city have been in the Red zone because of a Covid-19 patient, Nurul Iman mosque has been carrying out congregational worship while still applying the principle of *ḥifẓ al-nafs* (protection of life). Affirming the earlier work carried out by Mudzhar (2013), Nurul Iman mosque has basically carried out the *ijtihād* movement in carrying out the dialectic between efforts to save human life and carry out worship.

The spread of covid-19 in the city of Bengkulu has demanded a disciplined lifestyle for mosque residents, mosque administrators, mosque imam or mosque congregants in applying the principle of *ḥifẓ al-nafs* (protection of life), namely conducting congregational prayers by shortening the period of worship, maintaining a safe distance among worshipers, wearing of masks, ensuring the environment of the place of worship is clean and healthy, ensuring the individual's condition of physical and spiritual hygiene, as well as ensuring that the body is in a healthy condition.

Even though the city of Bengkulu has been designated as red zone by the Governor of Bengkulu on the 31^st^ March 2020, but the discipline in applying the principle of *ḥifẓ al-nafs* when carrying out these prayers and worshipping activities has been an important part of the success in maintaining the safety of mosque residents from potential covid-19 transmission. Enforcement of strict disciplines of health and safety protocols, which in Islamic terminology is known as the application of the principle of *ḥifẓ al-nafs* (protection of life) has been proven to be able to prevent the spread of covid-19 among residents of Nurul Iman mosque. This has also been supported by the research by Yuningsih (2020). Thus, residents of Nurul Iman mosque have organized worship practices that apply the *ḥifẓ al-nafs* principle (which is in accordance with the health protocol) which can be explained as follows:

First, the Imam of mosque recites shorter surah (chapters of verses in the holy Qur'an) in the implementation of congregational sholat prayers, both in the five daily prayers and in the Friday prayers while also shortening the Friday sermons. The recitations of shorter surah has been one of the efforts of imam of mosque to prevent the transmission of covid-19. Likewise, the mosque congregation also recite shorter Qur'anic surahs when they performed the Sunnah prayer, so that they can also reduce the period of meeting between congregations in the mosque. The habits of Imam and mosque congregation are in accordance with the recommendation of the fatwa of the Indonesian Ulema Council Number: 31 of 2020 which supported the practice of praying by reciting short surahs of al-Qur'an and also the implementation of shortened Friday sermons.

Second, the implementation of congregational prayer services was also carried out by maintaining distance between congregations. With the existence of covid-19 pandemic, the implementation of congregational prayers was no longer recommended to compile a meeting row. Under the conditions of Covid-19 pandemic, congregational worship is to be performed by maintaining physical distance among congregants, while upholding the virtue and requirements of congregational prayer. Corroborating this principle, Kurniawan (2020), Putra (2020), and Zhang et al. (2020) stated that within an emergency situation is urgent such as the emergency of the spread of Covid-19, mosque congregations could keep their space from each other. More importantly, the fatwa (religious edict) of the Indonesian Ulema Council Number: 31 of 2020 supported the further spacing of the *shaf* (the lines of sholat prayers) to prevent the transmission of the COVID-19 epidemic.

Third, mosque congregants use masks when performing sholat prayers at mosque. The use of masks is part of preventing the transmission of covid-19. In Islamic legal norms, the use of masks covering both nose and mouth is not permissible during sholat prayers as this will prevent certain parts of the body to touch the ground. However, as Islam is a principle-centred dynamic way of life, it creates spaces of effective responses to everyday life as well as contingencies and emergencies. In this context, the essence of *ḥifẓ al-nafs* provides an adaptive and responsive principle to a health emergency such as the Covid-19 pandemic. This principle of protection of life supercedes Islamic legal norms allowing and indeed imploring congrants of mosque to wear masks while still performing the sholat prayers. This is in accordance to the fatwa of the Indonesian Ulema Council Fatwa Number: 31.

Fourth, *ḥifẓ al-nafs* also upholds mental health as part of the protection of life. Indeed, maintaining and enhancing mental health is crucially important during the Covid-19 pandemic. As high levels of anxiety can compromise physical health, thus the Imam through his religious sermons and teachings as well as informal sessions of dialogue and discussions, provides guidance and reminders for members of the congregation to be more conscious, patience and steadfast in facing and fighting with the threat of the Covid-19 pandemic. Indeed, the congregations reported became more alert, patient and resolute due to the confidence-building efforts and the comforting care of the mosque's leadership. In strengthening this mental-emotional capacity, the Nurul Iman mosque has held joint sholat sessions to pray for God's protection against the spread of Covid-19 in their community. In a separate research, Hanif Muzaqi et al. (2020) reported similar findings highlighting the assistance of religious leaders' important role in encouraging increased awareness of community members to adapt to new norms including the living tradition in the Covid-19 pandemic era.

Fifth, the activity of purifying (*ṭaharah*) from all forms of najis (impurities), minor and major hadaṡ (condition requiring purification) has become an integral part of Nurul Iman mosque. It is also being interpreted and habitualized well by the mosque community as a form of physical and spiritual cleansing and purifying of both body and soul. In Islamic religious norms, acts of purification (*ṭaharah*) are obligatory requirements prior to vital worshipping activities for Muslims, especially but not only sholat prayers. This is propagated in the very beginning of the teachings in the books of *fiqh* (Islamic laws and obligatory rituals). As stated in research works of Tabi'in (2020), these activities of purification *(ṭaharah*) have also proven to be a part of the effectiveness in preventing the transmission of covid-19, whom also stated that a hygienic lifestyle can actually avert such viral spreading of diseases.

## 5 Conclusions

In reaffirming the contention of this article, the realization of the *hifẓ al-nafs* as a principle to protect human life has been demonstrated by the mosque congragations. In so doing, all the combined acts of *hifẓ al-nafs* -guided worshipping ranging from purifying rituals *(ṭaharah*) from all forms of impurities, either minor and major *hadaṡ*, to shortening of prayer and *dhikr*, to cleaning and sanitizing the mosque internal and external environment, to mandating the wearing of masks and usage of own prayer mat brought from home by mosque congragants have contributed to the prevention of Covid-19 transmission and simultaneously meeting the criteria of government's health protocols. Therefore, guided by the Islamic framework of the levels or types of need, the application of *hifẓ al-nafs* principle (protection of life), translated and operationalized into health protocols in attending, worshipping in, caring for the mosque is a primary necessity (*ḍarūriyyāt*). The application of *hifẓ al-nafs*, under Covid-19, is not a secondary need (*hājiyyāt*) or a complementary need (*taḥsīniyyāt*).

Based on this contention, this study proposes that the implementation of this combined series of Islamic rituals in mosques elsewhere in Indonesia and perhaps beyond should be carried out based on the principles of *hifẓ al-nafs* while contextualizing to realities in each community. It is towards the higher aims of public health and the sanctity of life in this world and the hereafter as cherished by Muslims that the application of *hifẓ al-nafs* is both urgent and important.

## Declarations

### Author contribution statement

Moh Dahlan: Conceived and designed the experiments; Performed the experiments; Analyzed and interpreted the data; Contributed reagents, materials, analysis tools or data; Wrote the paper.

Mohammad Reevany Bustami: Analyzed and interpreted the data; Wrote the paper.

Makmur: Conceived and designed the experiments; Performed the experiments; Wrote the paper.

Siti Mas'ulah: Contributed reagents, materials, analysis tools or data; Wrote the paper.

### Funding statement

This research did not receive any specific grant from funding agencies in the public, commercial, or not-for-profit sectors.

### Data availability statement

Data included in article.

### Declaration of interests statement

The authors declare no conflict of interest.

### Additional information

No additional information is available for this paper.
